# The joint role of systemic immune-inflammation index and geriatric nutritional risk index in cancer survivors and their impact on all-cause mortality

**DOI:** 10.3389/fnut.2025.1587824

**Published:** 2025-06-27

**Authors:** Xiangrui Chen, Min Hu, Chengluo Hao, Jun Li, Yunwei Han

**Affiliations:** ^1^Department of Oncology, The Affiliated Hospital of Southwest Medical University, Luzhou, China; ^2^Department of Oncology, Third People’s Hospital of Zigong, Zigong, China; ^3^Department of Dermatology, Third People’s Hospital of Zigong, Zigong, China

**Keywords:** systemic immune-inflammation index, geriatric nutritional risk index, cancer survivors, all-cause mortality, Dietary Inflammatory Index, NHANES

## Abstract

**Introduction:**

This study aimed to investigate the combined predictive value of the Systemic Immune-Inflammation Index (SII) and the Geriatric Nutritional Risk Index (GNRI) for all-cause mortality in cancer survivors.

**Methods:**

Using NHANES data (1999–2018), 2,969 eligible cancer survivors were categorized into four groups based on SII and GNRI levels. Mortality risk was assessed through unadjusted and fully adjusted Cox proportional hazards models.

**Results:**

The combination of low SII and high GNRI was associated with the lowest mortality risk (HR = 1.0, reference). In contrast, high SII and low GNRI significantly increased mortality risk (fully adjusted HR = 6.178, 95% CI: 2.669–14.299). Both unadjusted and adjusted models confirmed that high SII correlated with higher mortality, while low GNRI independently predicted poorer outcomes. Subgroup analyses revealed significant interactions between the SII-GNRI combination and gender/alcohol consumption.

**Discussion:**

The findings highlight SII and GNRI as critical predictors of all-cause mortality in cancer survivors. Their combined assessment may improve risk stratification and guide targeted clinical interventions.

## 1 Introduction

Cancer remains a significant global health challenge, and as the number of cancer survivors continues to grow, the demand for comprehensive survivorship care is also increasing (1). Older cancer survivors face greater survival challenges due to issues such as malnutrition and inflammation, which are closely linked to poorer survival outcomes (2–4). Recent studies highlight the critical role of nutrition and inflammation management in survivorship care. These modifiable factors can help alleviate metabolic dysfunction and immune suppression, which are key mechanisms driving poor outcomes in cancer survivors (5, 6). A deeper understanding of the interactions between nutrition and inflammation is essential for developing effective intervention strategies to improve long-term survival and quality of life.

The Systemic Immune-Inflammation Index (SII, platelets count × neutrophil/lymphocyte ratio) is a comprehensive inflammation marker defined as the platelet count multiplied by the neutrophil-to-lymphocyte ratio (7). Platelets can promote cancer cell growth and metastasis in the tumor microenvironment; neutrophils, as key participants in inflammation, can suppress anti-tumor immune responses; lymphocytes play a crucial role in anti-tumor immunity. SII has been widely used to assess inflammation in various diseases, particularly in cancer patients, where it is significantly associated with poor prognosis (8, 9). By integrating multiple blood components, SII can comprehensively reflect the body’s inflammation and immune status. High-SII levels may indicate severe inflammation, which is closely related to immune escape and tumor progression in the tumor microenvironment. Chronic inflammation and immune dysregulation are important mechanisms in cancer development, and increased SII often predicts poorer clinical outcomes, including higher all-cause mortality in cancer patients (10).

Nutritional status is a key factor influencing survival outcomes in cancer survivors, particularly among older adults who face an increased risk of malnutrition (11, 12). The Geriatric Nutritional Risk Index (GNRI) is a well-established tool that assesses nutritional risk by combining serum albumin levels with body weight (13–15). GNRI has proven to be highly reliable in predicting survival outcomes, especially in hospitalized patients and those undergoing cancer treatment. For example, GNRI has been shown to predict surgical outcomes and overall survival rates in individuals with lung cancer (16–19). Lower GNRI scores are linked to higher recurrence rates and poorer survival in gastrointestinal cancers. Compared to other nutritional assessment tools like the Prognostic Nutritional Index (PNI) or the Subjective Global Assessment (SGA), GNRI presents notable advantages (20, 21). While PNI includes both serum albumin and total lymphocyte count, GNRI places more emphasis on physical parameters, such as body weight, which may be more directly indicative of nutritional risk, especially in the elderly (22). The impact of SII and nutritional status on the prognosis of cancer survivors remains insufficiently explored. GNRI, based on objective measures like serum albumin and body weight, is particularly practical for use in outpatient settings. Moreover, it provides a comprehensive assessment of both nutritional and inflammatory status, making it highly relevant for cancer survivorship care.

The combined assessment of the SII and the GNRI holds significant importance in prognosis research for cancer survivors. Firstly, as an effective indicator of inflammatory and immune status, the SII can reflect information such as the quantity of immune cells in the body and the circulating blood volume. This, in turn, demonstrates the balance between inflammation and immunity within the body. On the other hand, the GNRI primarily reflects an individual’s nutritional status and has high clinical value in evaluating patients’ nutritional risks and nutritional levels. Additionally, the Dietary Inflammatory Index (DII) reflects the overall inflammatory potential of an individual’s diet, with a higher DII score indicating a more pro-inflammatory diet that may exacerbate systemic inflammation and negatively impact nutritional status (23). Secondly, there is a close interaction between inflammation and nutritional status in cancer survivors. When the level of inflammation rises, it triggers a series of physiological changes. For example, it increases the body’s energy consumption and disrupts the normal functions of the gastrointestinal tract. These changes may significantly increase the risk of malnutrition. Conversely, malnutrition further impairs immune system function, reducing the body’s ability to resist diseases and tumor progression, thus forming a vicious cycle.

Based on the inflammation-nutrition interaction mechanism, the combined assessment of SII and GNRI provides a comprehensive evaluation of cancer survivors’ health from two crucial physiological dimensions, enabling more accurate prognosis risk assessment and personalized intervention strategies to break the inflammation-nutrition vicious cycle, ultimately improving long-term survival and quality of life; this study examines the relationship between SII and GNRI with mortality in cancer survivors, analyzing their combined effects to offer evidence-based recommendations for integrating these modifiable factors into clinical survivorship care.

The findings aim to provide evidence-based recommendations for incorporating these modifiable factors into clinical survivorship care.

## 2 Materials and methods

### 2.1 Study population

The National Health and Nutrition Examination Survey (NHANES), conducted by the National Center for Health Statistics (NCHS), is a cross-sectional survey aimed at assessing the health and nutritional status of the United States population (24, 25). NHANES uses a complex, stratified, multistage probability sampling design to ensure the representation of national samples. A comprehensive description of its methodology and protocols is available in the existing literature. The data collection process includes interviews on demographics, diet, and health, as well as physical examinations and laboratory tests conducted in mobile examination centers. All NHANES protocols received approval from the NCHS Research Ethics Review Board, and written informed consent was obtained from all participants.

While NHANES is primarily a cross-sectional survey, it also supports longitudinal analysis through linkage with the National Death Index (NDI), enabling researchers to investigate long-term survival outcomes. This study utilized NHANES data from 1999 to 2018, linked with NDI mortality data. Participants aged 40 years or older were included, and sociodemographic characteristics, health status, lifestyle factors, and daily physical activity levels were analyzed to explore the associations between physical activity, nutritional status, and survival outcomes in cancer survivors. Mortality data for this study were obtained from the NCHS mortality files, linked to the NDI, as of December 31, 2019.

### 2.2 Definition of SII

Previous studies have shown that the SII is a comprehensive inflammation marker calculated as SII = (platelet count × neutrophil count)/lymphocyte count, expressed in units of × 10^∧^9 cells/μl. Complete blood counts were performed using a Coulter^®^ DxH 800 analyzer, supervised by a laboratory technician.

### 2.3 GNRI assessment

Nutritional status was evaluated using the GNRI, a validated tool initially created to assess nutritional risk in older patients within hospital or clinical environments. The GNRI has been validated in relation to complications in elderly populations. It is designed to identify individuals at risk of malnutrition-related issues by combining serum albumin levels and ideal body weight, which are both indicators of nutritional status. However, its main purpose is as a risk assessment tool rather than a comprehensive nutritional evaluation. The GNRI specifically focuses on capturing malnutrition-related risk. The GNRI was calculated using the following formula: GNRI = (1.489 × serum albumin (g/L)) + (41.7 × actual weight (kg)/ideal weight (kg)). Actual weight refers to the weight currently measured (whether self-reported, directly measured, or obtained from medical records). Ideal weight is calculated using a standard BMI of 22 kg/m^2^ with the formula: Ideal weight (kg) = 22 × (height (m)^2^). In this study, GNRI scores were classified as high risk (≤98) and low risk (>98), consistent with previous research and clinical guidelines. While originally developed for hospitalized older adults, GNRI has been validated in various populations, including community-dwelling individuals and epidemiological cohorts, confirming its usefulness in assessing nutritional risk and survival outcomes in older cancer survivors (26, 27).

Based on the SII (× 10∧9 cells/μl) and the GNRI, participants were classified into four groups: (1) Low-SII and High-GNRI group: SII < 1200, GNRI ≤ 98; (2) Low-SII and Low-GNRI group: SII < 1200, GNRI > 98; (3) High-SII and High-GNRI group: SII ≥ 1200, GNRI ≤ 98; and (4) High-SII and Low-GNRI group: SII ≥ 1200, GNRI > 98. This classification enabled analysis of the combined effects of SII and nutritional status on health outcomes.

### 2.4 Covariate inclusion

We included covariates in the multivariable analysis to adjust for potential confounders: demographic characteristics (age, sex, race/ethnicity, education level, family income), health behaviors (smoking status, alcohol consumption), nutritional status as measured by the DII, and clinical characteristics [body mass index (BMI), diabetes, hypertension]. For covariates with missing data, we employed mode imputation and random forest imputation methods. Detailed data descriptions can be found in the Supplementary material.

### 2.5 Statistical analysis methods

Participants in this study were categorized based on SII and GNRI. Descriptive statistics included means ± standard deviations for normally distributed continuous variables (analyzed using one-way ANOVA), medians (interquartile ranges) for non-normally distributed variables (analyzed using the Kruskal-Wallis test), and weighted proportions (%) for categorical variables (analyzed using design-adjusted chi-square tests). Differences between groups were assessed using *p*-values, with *p* < 0.05 indicating statistically significant differences.

Logistic regression was used to evaluate the associations between SII, GNRI, and all-cause mortality outcomes in cancer patients. Three models were constructed: a crude (unadjusted) model, Model 1 (adjusted for age, sex, and race), and a fully adjusted Model 2 (further adjusted for education, income, BMI, smoking, alcohol consumption, hypertension, diabetes, and DII). Cox proportional hazards regression was used to evaluate the associations between the DII and all-cause mortality outcomes in cancer survivors. Three models were constructed: a non-adjusted model (adjusting for none), Adjust I model (adjusted for gender, age, and race), and an adjusted Model II (further adjusted for poverty-income ratio, education, BMI, diabetes, drinking, hypertension, and smoking).

To better determine the impact of various factors on outcomes across different groups and their interactions, we conducted interaction tests (P Interaction) to assess the interaction effects between different factors and the combination of SII and GNRI.

## 3 Results

### 3.1 Baseline characteristics of participants

**FIGURE 1:**
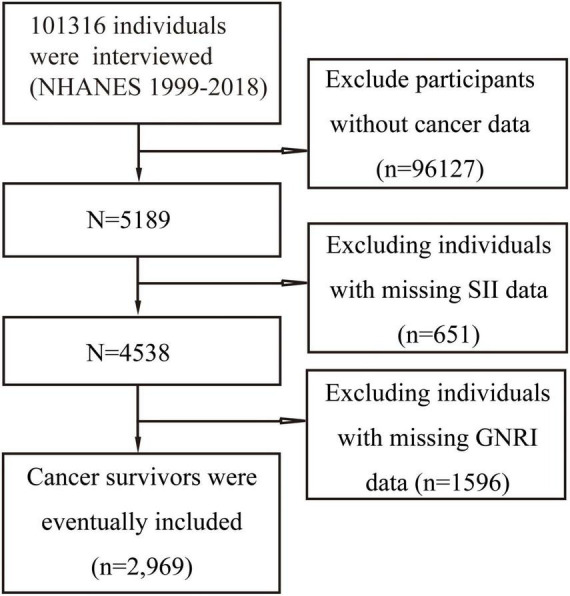
Flowchart of participant selection.

After screening 10 cycles of NHANES data in this study, 2,969 individuals met the inclusion criteria and were ultimately included in the analysis. The specific screening process is detailed in Figure 1. All participants were divided into four groups based on SII and GNRI: low-SII and high-GNRI group, high-SII and high-GNRI group, low-SII and low-GNRI group, and high-SII and low-GNRI group. Significant differences were observed in baseline characteristics among the groups (*P* < 0.05).

The low-SII and high-GNRI group (*n* = 2,633) had an average age of 65.002 ± 14.251 years, a BMI of 29.595 ± 6.439 kg/m^2^, with 47.246% males and 52.754% females. The high-SII and high-GNRI group (*n* = 133) had an average age of 67.053 ± 13.112 years, a BMI of 30.006 ± 7.499 kg/m^2^, with 52.632% males and 47.368% females. The low-SII and low-GNRI group (*n* = 165) had an average age of 70.388 ± 14.348 years, a BMI of 19.926 ± 2.470 kg/m^2^, with 36.970% males and 63.030% females. The high-SII and low-GNRI group (*n* = 38) had an average age of 76.816 ± 10.311 years, a BMI of 21.043 ± 2.951 kg/m^2^, with 47.368% males and 52.632% females. In terms of racial distribution, the White population had the highest proportion (66.844% in the low-SII and high-GNRI group, 73.684% in the high-SII and high-GNRI group, 68.485% in the low-SII and low-GNRI group, and 84.211% in the high-SII and low-GNRI group). Regarding education level, the high education group had the highest proportion (57.311% in the low-SII and high-GNRI group, 52.632% in the high-SII and high-GNRI group, 52.727% in the low-SII and low-GNRI group, and 57.895% in the high-SII and low-GNRI group). In terms of comorbidities, the prevalence of hypertension was 56.893% in the low-SII and high-GNRI group, 70.677% in the high-SII and high-GNRI group, 44.848% in the low-SII and low-GNRI group, and 57.895% in the high-SII and low-GNRI group. The prevalence of diabetes was 20.281% in the low-SII and high-GNRI group, 27.820% in the high-SII and high-GNRI group, 12.121% in the low-SII and low-GNRI group, and 7.895% in the high-SII and low-GNRI group. Smoking and alcohol consumption also showed significant differences among the groups (*P* < 0.05).

For all-cause mortality, the rates were 21.003% in the low-SII and high-GNRI group, 42.857% in the high-SII and high-GNRI group, 61.212% in the low-SII and low-GNRI group, and 76.316% in the high-SII and low-GNRI group. In terms of cancer mortality, there were significant differences among groups (*p* < 0.001), with the high-SII and low-GNRI group having the highest proportion of cancer deaths (26.316%) compared to 7.026% in the low-SII and high-GNRI group. The detailed distribution was as follows: 114 cancer deaths (85.714%) in the high-SII and high-GNRI group; 32 cancer deaths (19.394%) in the low-SII and low-GNRI group; 10 cancer deaths (26.316%) in the high-SII and low-GNRI group. Notably, the DII showed significant differences across groups, with the low-SII and high-GNRI group having the lowest mean DII score (1.140 ± 1.925) compared to the high-SII and low-GNRI group with the highest (1.385 ± 1.878, *p* = 0.480). Table 1 summarizes the detailed characteristics of the patient population across the four different SII and GNRI level groups.

**TABLE 1:** Comparison of demographic and health characteristics across SII and GNRI groups.

Variable	Low-SII and high-GNRI group	High-SII and high-GNRI group	Low-SII and low-GNRI group	High-SII and low-GNRI group	*P*-value
*N*	2633	133	165	38	
Age, years	65.002 ± 14.251	67.053 ± 13.112	70.388 ± 14.348	76.816 ± 10.311	<0.001
Poverty income ratio	2.744 ± 1.546	2.710 ± 1.535	2.600 ± 1.478	2.685 ± 1.423	0.694
BMI, kg/m2	29.595 ± 6.439	30.006 ± 7.499	19.926 ± 2.470	21.043 ± 2.951	<0.001
Gender, *n* (%)					0.038
Male	1244 (47.246%)	70 (52.632%)	61 (36.970%)	18 (47.368%)	
Female	1389 (52.754%)	63 (47.368%)	104 (63.030%)	20 (52.632%)	
Race, *n* (%)					0.078
Mexican	191 (7.254%)	10 (7.519%)	5 (3.030%)	2 (5.263%)	
Hispanic	179 (6.798%)	6 (4.511%)	6 (3.636%)	1 (2.632%)	
White	1760 (66.844%)	98 (73.684%)	113 (68.485%)	32 (84.211%)	
Black	359 (13.635%)	13 (9.774%)	32 (19.394%)	3 (7.895%)	
Other Race	144 (5.469%)	6 (4.511%)	9 (5.455%)	0 (0.000%)	
Education status, *n* (%)					0.192
Low	530 (20.129%)	34 (25.564%)	44 (26.667%)	11 (28.947%)	
Medium	594 (22.560%)	29 (21.805%)	34 (20.606%)	5 (13.158%)	
High	1509 (57.311%)	70 (52.632%)	87 (52.727%)	22 (57.895%)	
Diabetes, *n* (%)					0.002
No	2099 (79.719%)	96 (72.180%)	145 (87.879%)	35 (92.105%)	
Yes	534 (20.281%)	37 (27.820%)	20 (12.121%)	3 (7.895%)	
Drinking, *n* (%)					<0.001
≤1 drink/day	1483 (56.324%)	84 (63.158%)	127 (76.970%)	32 (84.211%)	
>1 drink/day	1150 (43.676%)	49 (36.842%)	38 (23.030%)	6 (15.789%)	
Hypertension, *n* (%)					<0.001
No	1135 (43.107%)	39 (29.323%)	91 (55.152%)	16 (42.105%)	
Yes	1498 (56.893%)	94 (70.677%)	74 (44.848%)	22 (57.895%)	
Smoke, *n* (%)					0.023
Never	1229 (46.677%)	52 (39.098%)	66 (40.000%)	13 (34.211%)	
Former	1000 (37.979%)	54 (40.602%)	59 (35.758%)	17 (44.737%)	
Current	404 (15.344%)	27 (20.301%)	40 (24.242%)	8 (21.053%)	
All-cause Mortality, n (%)					<0.001
No	2080 (78.997%)	76 (57.143%)	64 (38.788%)	9 (23.684%)	
Yes	553 (21.003%)	57 (42.857%)	101 (61.212%)	29 (76.316%)	
Cancer Mortality, n (%)					<0.001
No	2448 (92.974%)	114 (85.714%)	133 (80.606%)	28 (73.684%)	
Yes	185 (7.026%)	19 (14.286%)	32 (19.394%)	10 (26.316%)	
SII, *n* (%)					<0.001
Low	2633 (100.000%)	0 (0.000%)	165 (100.000%)	0 (0.000%)	
High	0 (0.000%)	133 (100.000%)	0 (0.000%)	38 (100.000%)	
GNRI, *n* (%)					<0.001
High	0 (0.000%)	0 (0.000%)	165 (100.000%)	38 (100.000%)	
Low	2633 (100.000%)	133 (100.000%)	0 (0.000%)	0 (0.000%)	
DII, *n* (%)	1.140 ± 1.925	1.385 ± 1.878	1.248 ± 1.934	1.108 ± 1.773	0.480

### 3.2 Correlation between SII, GNRI and mortality

**FIGURE 2:**
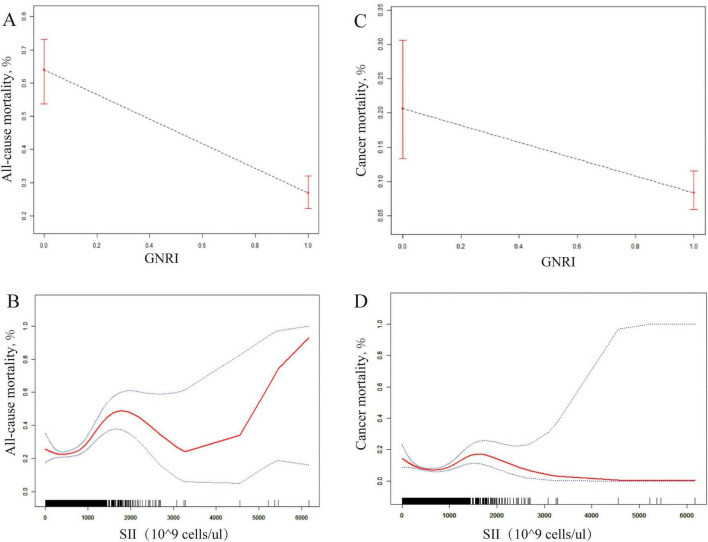
Comparative Dose-Response analysis of GNRI and SII as prognostic biomarkers for post-cancer survival. **(A)** (GNRI vs all-cause mortality): Linear decrease in risk with higher GNRI (HR 0.82 per 1-unit increase, 95% CI 0.79–0.85). **(B)** (SII vs all-cause mortality): Risk rises sharply when SII >3,000 cells/μL. **(C)** (GNRI vs cancer mortality): Consistent protective effect (HR 0.89, 95% CI 0.84–0.94). **(D)** (SII vs cancer mortality): Biphasic pattern: neutral (<1,500 cells/μL), modest risk (1,500–3,000 cells/μL; HR 1.12), high risk (>3,000 cells/μL; HR 1.58).

Among 2,969 cancer survivors, a total of 740 all-cause deaths and 352 cancer-specific deaths occurred during follow-up. Figure 2 illustrates the dose-response relationships between SII, GNRI, and mortality endpoints. For all-cause mortality, the scatter plot (Panel A) reveals a significant negative correlation between GNRI and mortality risk (adjusted HR per 1-unit increase: 0.82, 95% CI 0.79–0.85), while the fitted curve (Panel B) demonstrates a non-linear threshold effect for SII, with increased risk observed at values >3,000 cells/μl. In contrast, cancer mortality analysis (Panels C/D) shows that although GNRI maintains a protective effect (HR 0.89, 95% CI 0.84–0.94), the SII-mortality association follows a biphasic pattern - low SII (<1,500 cells/μl) exhibits neutral risk, intermediate levels (1,500–3,000 cells/μl) show modest associations (HR 1.12, 95% CI 1.01–1.24), and high SII (>3,000 cells/μl) significantly increases cancer mortality risk (HR 1.58, 95% CI 1.21–2.07).

**FIGURE 3:**
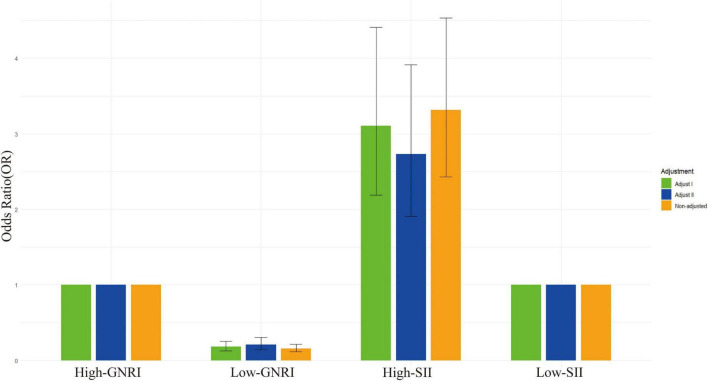
Odds ratios (OR) for different GNRI and SII levels with various adjustments.

In the regression analysis (Table 2), the unadjusted model results show that cancer survivors with high-SII had a hazard ratio (HR) of 3.315 (95% CI: 2.426, 4.531) for all-cause mortality, indicating a significantly increased risk and suggesting that high-SII is associated with a higher risk of death (Figure 3). Meanwhile, cancer survivors with low-GNRI had an HR of 0.159 (95% CI: 0.118, 0.215), indicating a significantly reduced risk and suggesting that low-GNRI is associated with a lower risk of death. In the fully adjusted model (Model 2), which accounted for potential confounders such as sex, age, race, education level, poverty-to-income ratio, BMI, diabetes, alcohol consumption, hypertension, smoking, and DII, the results still showed that cancer survivors with high-SII had an HR of 2.728 (95% CI: 1.903, 3.911) for all-cause mortality, and those with low-GNRI had an HR of 0.207 (95% CI: 0.141, 0.304). These findings indicate that, both in unadjusted and fully adjusted models, high-SII and low-GNRI significantly influence all-cause mortality in cancer survivors, with high-SII associated with a higher risk of death and low-GNRI associated with a lower risk of death.

**TABLE 2:** Risk ratios for all-cause mortality across SII and GNRI groups.

Variable	Non-adjusted	Adjust I	Adjust II
**SII**			
Low	1.0	1.0	1.0
High	3.315 (2.426, 4.531) < 0.00001	3.105 (2.187, 4.406) < 0.00001	2.728 (1.903, 3.911) < 0.00001
**GNRI**			
High	1.0	1.0	1.0
Low	0.159 (0.118, 0.215) < 0.00001	0.181 (0.128, 0.254) < 0.00001	0.207 (0.141, 0.304) < 0.00001
**SII and GNRI**			
Low-SII and high-GNRI group	1.0	1.0	1.0
High-SII and high-GNRI group	2.820 (1.975, 4.025) < 0.00001	2.950 (1.987, 4.381) < 0.00001	2.669 (1.784, 3.995) < 0.00001
Low-SII and low-GNRI group	5.933 (4.279, 8.227) < 0.00001	5.693 (3.918, 8.272) < 0.00001	4.925 (3.251, 7.461) < 0.00001
High-SII and low-GNRI group	12.114 (5.701, 25.741) < 0.00001	7.001 (3.093, 15.849) < 0.00001	6.176 (2.668, 14.296) 0.00002

Non-adjusted model: No adjustment for potential confounders. Adjust I model: Adjusted for gender, age, and race. Adjust II model: Adjusted for gender, age, race, education level, poverty-to-income ratio, BMI, diabetes, alcohol consumption, hypertension, smoking, and DII.

Similarly, in the analysis of cancer-specific mortality (Table 3), the unadjusted model revealed that high-SII was significantly associated with an increased risk (HR 2.429, 95% CI: 1.592–3.707), while low-GNRI was linked to a reduced risk (HR 0.305, 95% CI: 0.211–0.441). Even after adjusting for confounders in the fully adjusted model, high-SII remained a significant predictor (HR 2.000, 95% CI: 1.288–3.107), and low-GNRI continued to show a protective effect (HR 0.350, 95% CI: 0.223–0.548). Notably, combinations of high-SII and low-GNRI exhibited the strongest associations, with the high-SII/low-GNRI group showing a fourfold elevated risk (HR 4.726, 95% CI: 2.261–9.879 in the unadjusted model; HR 3.378, 95% CI: 1.523–7.495 adjusted). These findings underscore the dual prognostic significance of SII and GNRI—high SII as a risk factor and low GNRI as a protective factor—for both all-cause and cancer-specific mortality in survivors.

**TABLE 3:** Risk ratios for cancer mortality across SII and GNRI groups.

Variable	Non-adjusted	Adjust I	Adjust II
**SII**			
Low	1.0	1.0	1.0
High	2.429 (1.592, 3.707) 0.00004	2.175 (1.413, 3.349) 0.00042	2.000 (1.288, 3.107) 0.00203
**GNRI**			
High	1.0	1.0	1.0
Low	0.305 (0.211, 0.441) < 0.00001	0.352 (0.239, 0.516) < 0.00001	0.350 (0.223, 0.548) < 0.00001
**SII and GNRI**			
Low-SII and high-GNRI group	1.0	1.0	1.0
High-SII and high-GNRI group	2.205 (1.327, 3.666) 0.00228	2.100 (1.253, 3.519) 0.00484	1.983 (1.175, 3.347) 0.01032
Low-SII and low-GNRI group	3.184 (2.105, 4.816) < 0.00001	2.873 (1.869, 4.416) < 0.00001	2.897 (1.774, 4.730) 0.00002
High-SII and low-GNRI group	4.726 (2.261, 9.879) 0.00004	3.459 (1.622, 7.375) 0.00132	3.378 (1.523, 7.495) 0.00275

Non-adjusted model: No adjustment for potential confounders. Adjust I model: Adjusted for gender, age, and race. Adjust II model: Adjusted for gender, age, race, education level, poverty-to-income ratio, BMI, diabetes, alcohol consumption, hypertension, smoking, and DII.

### 3.3 Correlation between combined SII and GNRI and mortality

In the joint analysis, we evaluated the combined effects of SII and GNRI on all-cause mortality. In the non-adjusted model presented in Table 2, the group with low-SII (SII < 1200 × 10^9^ cells/μl) and high-GNRI (GNRI > 98) was used as the reference group, having a hazard ratio (HR) of 1.0. This group was associated with the lowest risk of all-cause mortality. When looking at other groups, the high-SII and high-GNRI group had a significantly increased risk. In the non-adjusted model, the HR was 2.820 (95% CI: 1.975, 4.025; *P* < 0.001), which decreased slightly to 2.950 (95% CI: 1.987, 4.381; *P* < 0.001) in the Adjust I model (adjusted for gender, age, and race), and further reduced to 2.669 (95% CI: 1.784, 3.995; *P* < 0.001) in the Adjust II model (adjusted for multiple factors including gender, age, race, education level, poverty-to-income ratio, BMI, diabetes, alcohol consumption, hypertension, smoking, and DII).

**FIGURE 4:**
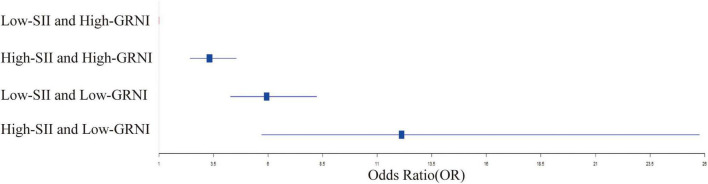
Forest plot of odds ratios (OR) for different SII and GNRI combinations.

The low-SII and low-GNRI group showed an even greater increase in the risk of all-cause mortality. The unadjusted HR was 5.933 (95% CI: 4.279, 8.227; *P* < 0.001), 5.693 (95% CI: 3.918, 8.272; *P* < 0.001) in the Adjust I model, and 4.925 (95% CI: 3.251, 7.461; *P* < 0.001) in the Adjust II model. Most notably, the high-SII and low-GNRI group had the highest risk of all-cause mortality. The unadjusted HR was an extremely high value of 12.114 (95% CI: 5.701, 25.741; *P* < 0.001), which decreased to 7.001 (95% CI: 3.093, 15.849; *P* < 0.001) in the Adjust I model and further declined to 6.176 (95% CI: 2.668, 14.296; *P* < 0.001) in the Adjust II model. These data clearly show that the combination of low-SII and high-GNRI is associated with the lowest risk of all-cause mortality, while the combination of high-SII and low-GNRI significantly increases the risk of all-cause mortality (Figure 4). The adjusted models further support this conclusion, highlighting the crucial role of SII and GNRI in predicting all-cause mortality.

Similar patterns were observed for cancer mortality. The low-SII and high-GNRI group still served as the reference group with an HR of 1.0. The high-SII and high-GNRI group had an unadjusted HR of 2.205 (95% CI: 1.327, 3.666; *P* = 0.002), which changed to 2.100 (95% CI: 1.253, 3.519; *P* = 0.005) in the Adjust I model and 1.983 (95% CI: 1.175, 3.347; *P* = 0.010) in the Adjust II model in Table 3. The low-SII and low-GNRI group had an unadjusted HR of 3.184 (95% CI: 2.105, 4.816; *P* < 0.001), 2.873 (95% CI: 1.869, 4.416; *P* < 0.001) in the Adjust I model, and 2.897 (95% CI: 1.774, 4.730; *P* < 0.001) in the Adjust II model. The high-SII and low-GNRI group had an unadjusted HR of 4.726 (95% CI: 2.261, 9.879; *P* < 0.001), 3.459 (95% CI: 1.622, 7.375; *P* = 0.001) in the Adjust I model, and 3.378 (95% CI: 1.523, 7.495; *P* = 0.003) in the Adjust II model. These findings in the cancer - specific mortality analysis further support the importance of considering the combined effects of SII and GNRI when predicting mortality outcomes in cancer survivors.

### 3.4 Correlation between DII and mortality

The hazard ratios for all-cause mortality associated with the DII varied significantly across subgroups defined by the combination of SII and GNRI (Table 4). In the non-adjusted model, the low-SII and high-GNRI group exhibited a marginally elevated risk (HR = 1.059, 95% CI: 1.015–1.106, *P* = 0.008), which persisted in adjusted models (HR = 1.060, 95% CI: 1.011–1.112, *P* = 0.015 in Adjust II). Conversely, the high-SII and high-GNRI group showed no significant association in either adjusted model (*P* > 0.05). The low-SII and low-GNRI group demonstrated increased risk in Adjust I (HR = 1.222, 95% CI: 1.070–1.395, *P* = 0.003), but this attenuated slightly in Adjust II (HR = 1.128, 95% CI: 0.980–1.298, *P* = 0.094). The high-SII and low-GNRI group presented the strongest association, with a markedly elevated HR in Adjust II (HR = 6.097, 95% CI: 1.714–21.691, *P* = 0.005), suggesting a potentially detrimental interaction between high inflammatory burden and poor nutritional status.

**TABLE 4:** Hazard ratios and 95% confidence intervals of DII associated with all-cause mortality by SII/GNRI subgroups.

Model	Non-adjusted	Adjust I	Adjust II
Low-SII and high-GNRI group	1.059 (1.015, 1.106) 0.00846	1.059 (1.012, 1.108) 0.01359	1.060 (1.011, 1.112) 0.01543
High-SII and high-GNRI group	0.926 (0.804, 1.068) 0.29058	0.941 (0.812, 1.091) 0.42194	0.985 (0.828, 1.172) 0.86894
Low-SII and low-GNRI group	1.132 (1.005, 1.275) 0.04168	1.222 (1.070, 1.395) 0.00315	1.128 (0.980, 1.298) 0.09447
High-SII and low-GNRI group	1.297 (1.010, 1.666) 0.04178	1.599 (1.091, 2.343) 0.01598	6.097 (1.714, 21.691) 0.00524
Total	1.056 (1.016, 1.097) 0.00565	1.070 (1.027, 1.114) 0.00110	1.065 (1.022, 1.111) 0.00295

Non-adjusted model: No adjustment for potential confounders. Adjust I model: Adjusted for gender, age, and race. Adjust II model: Adjusted for gender, age, race, education level, poverty-to-income ratio, BMI, diabetes, alcohol consumption, hypertension, and smoking.

The overall analysis confirmed a significant positive correlation between DII and all-cause mortality (Total model: HR = 1.065, 95% CI: 1.022–1.111, *P* = 0.003), even after comprehensive adjustment. Subgroup variations indicate that the prognostic impact of DII is context-dependent. Specifically, the protective effect observed in the high-SII and high-GNRI group (neutral association) contrasts with the heightened vulnerability in the high-SII and low-GNRI group.

### 3.5 Subgroup analysis and sensitivity analysis

The subgroup analysis results, as shown in Supplementary Table 2 in the Supplementary materials, indicate that gender and drinking have significant interaction effects with SII and GNRI (P Interaction = 0.048 and 0.072, respectively), suggesting that the effects of these variables may be moderated by SII and GNRI across different subgroups. Diabetes, hypertension, and smoking showed significant effects in all subgroups (*P*-values all < 0.01), but their interaction effects with SII and GNRI were not significant (*P* Interaction > 0.68), indicating that the impacts of these variables are relatively independent. Education had significant effects in some subgroups, but no significant interaction effects were observed (*P* Interaction = 0.577). Overall, gender and drinking may be key variables interacting with SII and GNRI, while other variables demonstrated independent and significant effects across subgroups (Supplementary Figure 1).

## 4 Discussion

This study analyzed 2,969 participants from 10 cycles of NHANES data, dividing them into four groups based on the SII and GNRI: low-SII and high-GNRI group, high-SII and high-GNRI group, low-SII and low-GNRI group, and high-SII and low-GNRI group. Baseline characteristics revealed significant differences (*P* < 0.05) among the groups in terms of age (ranging from 65.002 ± 14.251 years in the low-SII/high-GNRI group to 76.816 ± 10.311 years in the high-SII/low-GNRI group), BMI (ranging from 19.926 ± 2.470 kg/m^2^ in the low-SII/low-GNRI group to 30.006 ± 7.499 kg/m^2^ in the high-SII/high-GNRI group), gender distribution (47.2%–52.7% male across groups), race (predominantly White, with proportions ranging from 66.8% to 84.2%), education level (highest in the low-SII/high-GNRI group at 57.3%), and comorbidities (e.g., hypertension prevalence from 44.8% in the low-SII/low-GNRI group to 70.7% in the high-SII/high-GNRI group, and diabetes prevalence from 7.9% in the high-SII/low-GNRI group to 27.8% in the high-SII/high-GNRI group). All-cause mortality rates varied significantly across groups, with the low-SII and high-GNRI group exhibiting the lowest rate (21.0%) and the high-SII and low-GNRI group showing the highest rate (76.3%). Regression analysis demonstrated that high-SII was significantly associated with an increased risk of all-cause mortality (HR = 2.728, 95% CI: 1.903, 3.911), while low-GNRI was significantly associated with a reduced risk (HR = 0.207, 95% CI: 0.141, 0.304). Joint analysis further confirmed that the combination of low-SII and high-GNRI was associated with the lowest risk of all-cause mortality, whereas the combination of high-SII and low-GNRI significantly increased the risk (HR = 6.176, 95% CI: 2.668, 14.296 in the fully adjusted model). Subgroup analysis indicated that gender and alcohol consumption had significant interactions with SII and GNRI (P Interaction = 0.048 and 0.072, respectively), while diabetes, hypertension, and smoking showed independent and significant effects across all subgroups (*P* < 0.01 for all).

SII reflects systemic immune and inflammatory responses. Elevated SII levels are commonly associated with chronic inflammatory states, which may increase the risk of developing cardiovascular diseases, diabetes, and cancer, thereby elevating mortality rates (3, 28, 29). Chronic inflammation exacerbates organ dysfunction during aging, further heightening the risk of mortality. A study indicates that the SII is positively correlated with the risk of in-hospital mortality in patients with COPD, and SII can serve as an independent prognostic risk factor for this patient population (30). Another retrospective study found that the SII holds significant prognostic value in metastatic esophageal cancer, with elevated SII levels correlating with poorer survival outcomes (31). Other studies have found that the SII index has significant statistical value in predicting recurrence in laryngeal cancer patients, with a significantly higher recurrence rate observed in patients with high SII scores (32). GNRI, as an effective tool for assessing the nutritional status of the elderly, can effectively predict patient prognosis. A low GNRI indicates malnutrition, which is closely associated with impaired immune function, muscle loss, and increased risk of chronic diseases, further elevating the risk of mortality (33, 34). A retrospective cohort study demonstrated the significant prognostic value of GNRI in predicting mortality within 30 days and 365 days among patients with acute myocardial infarction. An increase in GNRI was associated with a notable decrease in mortality, highlighting the critical impact of nutritional status on the long-term survival of these patients (35). The DII, pioneered by Shivappa et al. quantifies the holistic inflammatory propensity of dietary patterns through a sophisticated algorithm that aggregates inflammatory response scores across 45 food parameters, weighted against six established biomarkers of systemic inflammation (IL-1β, IL-6, IL-10, TNF-α, CRP) (23). This metric establishes a standardized framework for examining diet-inflammation interactions, positing that dietary constituents modulate chronic inflammatory states via immunoregulatory pathways, thereby mediating pathophysiological risks. Empirical evidence substantiates a dose-dependent relationship between DII and mortality outcomes. Each one-unit increment in DII correlates with a 4% elevation in all-cause mortality risk (RR = 1.04; 95% CI: 1.03–1.05) and a 2% increase in cancer-specific mortality (RR = 1.02; 95% CI: 1.00–1.04), with statistical significance underscoring a robust linear association (36). Notably, the index’s public health significance resides in its capacity to synthesize synergistic effects embedded within complex dietary matrices—an analytical dimension transcending conventional single-nutrient paradigms (37). This integrative methodology enhances predictive precision, particularly in identifying high-risk subpopulations. DII as a pivotal metric for quantifying the inflammatory potential of dietary patterns, enables precise assessment of a diet’s pro-inflammatory and anti-inflammatory properties. DII as a pivotal metric for quantifying the inflammatory potential of dietary patterns, enables precise assessment of a diet’s pro-inflammatory and anti-inflammatory properties. This sophisticated index serves as an analytical cornerstone for dissecting the intricate interplay between dietary inflammatory status and SII, GNRI, as well as mortality risk trajectories. Moreover, it provides an evidence-based framework for refining nutritional intervention strategies tailored to cancer survivors, thereby optimizing clinical outcomes through personalized dietary modulation.

Possible reasons for the interaction between SII and GNRI: The activation of the immune system consumes substantial amounts of nutrients, especially during chronic inflammation or infection. Inflammatory markers such as CRP and TNF-α can suppress appetite, impairing protein and fat metabolism, which ultimately leads to malnutrition (38). Chronic inflammation exacerbates muscle catabolism and appetite suppression through pro-inflammatory cytokines such as IL-6 and TNF-α, leading to nutritional deterioration; in turn, malnutrition further impairs immune function and suppresses the production of anti-inflammatory factors, creating a positive feedback loop where “inflammation-driven nutrient depletion exacerbates nutritional deficits, which in turn intensify inflammation.” This vicious cycle synergistically accelerates cancer-related mortality (39). Malnutrition, in turn, weakens immune function, creating a vicious cycle. Adequate nutritional support is crucial for the proper functioning of the immune system. A high GNRI generally indicates better nutritional status, which enhances immune function and the ability to combat inflammation and infection. Conversely, a low GNRI suggests malnutrition, which may impair the immune response and increase disease risk (40, 41). A combined assessment of SII and GNRI offers a more comprehensive understanding of a patient’s health, as relying on either alone may not provide an accurate prediction of mortality risk.

The strengths of this study include its relatively large sample size (*N* = 2,969) from a nationally representative survey enhancing generalizability, novel evidence on non-linear SII/GNRI-mortality relationships through advanced statistical approaches, and robust subgroup validation; however, we acknowledge major limitations: the lack of tumor-specific data (type, stage, treatment) in NHANES prevents meaningful cancer subtype stratification which may exhibit distinct inflammation-nutrition interactions, the cross-sectional design restricts causal inference with potential residual confounding from unmeasured factors (treatment-related inflammation, disease-specific metabolic alterations), the representativeness may be compromised by United States-specific demographics and socioeconomic factors, and the small sample size in some subgroups (high-SII/low-GNRI group) may lead to unstable results, which necessitates validation in future research with expanded sample sizes; notably, our cancer sample reflects real-world prevalence but may have limited power for rare cancers, with some subgroups showing unstable effect estimates (e.g., high-SII/low-GNRI group HR 6.176, 95% CI 2.668–14.296); future research should prioritize cancer registry-linked cohorts with longitudinal data to address these issues, though we recognize this cross-sectional design provides initial valuable insights into potential prognostic biomarkers for cancer survivors. Current research indicates that the standardized thresholds of SII and GNRI vary significantly across different disease types (such as tumors, cardiovascular diseases, etc.). This heterogeneity poses challenges for clinicians in interpreting test results and makes it difficult to provide patients with precise health guidance based on unified indicators. It is recommended to integrate international multi - center cohorts (such as ENRICHD and other research cohorts covering multiple disease types and diverse geographical populations), pool data from individuals of different races and regions, and employ restricted cubic splines and dynamic panel models to clarify the non - linear relationship between SII and clinical outcomes. Ultimately, this will lead to the development of globally applicable clinical cut - off value guidelines.

## 5 Conclusion

This study demonstrates that the combination of low SII and high GNRI is associated with the lowest risk of all-cause mortality, while high SII and low GNRI significantly increase mortality risk. These findings suggest that SII and GNRI are promising but not definitive predictors of all-cause mortality in this population.

## Data Availability

Publicly available datasets were analyzed in this study. This data can be found here: https://wwwn.cdc.gov/nchs/nhanes/.
