# Supplementary material for: The joint role of systemic immune-inflammation index and geriatric nutritional risk index in cancer survivors and their impact on all-cause mortality
Source: Front Nutr. 2025 Jun 27;12:1587824. doi: 10.3389/fnut.2025.1587824 (PMC12245672; doi:10.3389/fnut.2025.1587824)
Supplement: Supplementary file 1 [file Data_Sheet_1.docx]

Supplementary Material

# Description of Covariates

Sex: Sex was categorized as male and female.

Age: Age was treated as a continuous variable and was not treated as a categorical variable.

Race: Race was categorized as non-Hispanic white, non-Hispanic black, Mexican American, and other races.

Education Level: Education levels are categorized into below high school, high school level, and above high school.

Household Income: The data used for household income is the ratio of household income to poverty (FPL). Household income was treated as a continuous variable and was not treated as a categorical variable.

BMI Status: Body mass index was calculated from measured height and weight as weight/height² (kg/m²), and it was treated as a continuous variable and not treated as a categorical variable.

Drinking Status: Drinking behavior was measured in the "Alcohol Use" questionnaire. In the "Alcohol Use" questionnaire, each respondent was asked how often they had drunk alcoholic drinks in the past 12 months. According to the question, participants were categorized into those who drank less than or equal to 1 drink/day and those who drank more than 1 drink/day.

Smoking status: Smoking behavior was measured in the “smoking: cigarette use” questionnaire. In the “smoking: cigarette use” questionnaire, respondents were asked if s/he had smoked at least 100 cigarettes in their life, and smoked cigarettes when being questioned. If the respondent had smoked less than 100 cigarettes in their life, s/he was classified as a never smoker. If the respondent had smoked at least 100 cigarettes in his/her life and still smoked when s/he answered the questionnaire, s/he was classified as a current smoker. The respondent was classified as a former smoker if s/he had smoked at least 100 cigarettes in his/her life, and had quit smoking when s/he answered the questionnaire. Smoking status was categorized into never smoker (reference group), former smoker, and current smoker.

Diabetes: Diabetes data were measured in the "Diabetes" questionnaire. In this questionnaire, respondents were asked if they had ever been told by a doctor or health professional that they have diabetes. The results were categorized into two groups: ever had diabetes or never had diabetes.

Hypertension: Data on hypertension were obtained through the "Blood Pressure & Cholesterol" questionnaire. In this questionnaire, respondents were asked if they had ever been told by a doctor or health professional that they have hypertension. The results were divided into two groups: those who have ever had hypertension and those who have never had hypertension.

Anemia: Anemia data were measured in the "Medical Conditions" questionnaire. In this questionnaire, respondents were asked if they had ever been on treatment for anemia during the past 3 months. The results were categorized into two groups: ever had anemia or never had anemia.

Dietary Inflammatory Index(DII): The data utilized for calculating the DII were derived from dietary intake information collected through the "Dietary Interview - Individual Foods" module of a comprehensive questionnaire survey. This module meticulously captured the types and quantities of all food and beverage items, encompassing all forms of water, consumed by participants within a precise 24-hour period spanning from midnight to midnight prior to the interview. Based on this detailed data, estimations were made regarding the intake of energy, nutrients, and various other food components. A total of 26 distinct food parameters were employed in the DII calculation, which included macronutrients such as carbohydrates, proteins, and total fats, along with specific nutrients like cholesterol, alcohol, dietary fiber, monounsaturated fatty acids (MUFA), saturated fatty acids, polyunsaturated fatty acids (PUFA), niacin, vitamin A, thiamine (vitamin B₁), riboflavin (vitamin B₂), vitamin B₆, vitamin B₁₂, vitamin C, vitamin D, vitamin E, and essential minerals including iron (Fe), zinc (Zn), and selenium (Se), magnesium (Mg). Additionally, water - soluble vitamins such as folate and β - carotene, a stimulant like caffeine, and overall energy intake were also incorporated. Following the Hébert protocol, the initial step involved the computation of Z - scores using the formula: Z - score = (the daily intake of a particular dietary component or nutrient - the global average daily intake) / the standard deviation of the global per - capita daily intake of that dietary component or nutrient. Subsequently, these Z - scores were transformed into percentiles. To achieve a symmetric distribution centered around "0", the obtained percentile values were multiplied by 2 and then subtracted by "1". Next, these adjusted percentile values were multiplied by the total inflammatory score of each dietary component to determine the inflammation index for each individual dietary component or nutrient. Finally, by summing up the inflammation indices of all 26 dietary component parameters, the overall DII was successfully calculated.

# Supplementary Table 1: Missing Covariates and Interpolation Methods

| **Covariate** | **Method for Missing Data** | **Missing Values** |
| --- | --- | --- |
| Household Income | Multiple imputation | 564 (9.53%) |
| BMI | Median imputation | 181 (3.06%) |
| Hypertension | Multiple imputation | 17 (0.29%) |
| Smoking | Multiple imputation | 20 (0.34%) |

# Supplementary Table 2: Hazard Ratios (HR) and Interaction P-values for Various Covariates

| **Variable** | **Low-SII & High-GNRI** | **High-SII & High-GNRI** | **Low-SII & Low-GNRI** | **High-SII & Low-GNRI** |
| --- | --- | --- | --- | --- |
| **Gender** | OR (95% CI): Ref. | 3.731 (2.181, 6.382) | 5.535 (2.865, 10.693) | 2.703 (0.930, 7.862) |
|  | P-value: - | <0.0001 | <0.0001 | 0.0679 |
|  | P Interaction: 0.0479 | 0.0479 | 0.0479 | 0.0479 |
| **Education** | OR (95% CI): Ref. | 2.896 (1.326, 6.322) | 5.610 (2.389, 13.174) | 4.246 (0.809, 22.294) |
|  | P-value: - | 0.0076 | <0.0001 | 0.0874 |
|  | P Interaction: 0.5766 | 0.5766 | 0.5766 | 0.5766 |
| **Diabetes** | OR (95% CI): Ref. | 2.563 (1.581, 4.154) | 4.877 (3.107, 7.657) | 7.544 (2.928, 19.437) |
|  | P-value: - | 0.0001 | <0.0001 | <0.0001 |
|  | P Interaction: 0.6882 | 0.6882 | 0.6882 | 0.6882 |
| **Drinking** | OR (95% CI): Ref. | 3.056 (1.838, 5.079) | 5.058 (3.034, 8.433) | 3.572 (1.495, 8.531) |
|  | P-value: - | <0.0001 | <0.0001 | 0.0042 |
|  | P Interaction: 0.0722 | 0.0722 | 0.0722 | 0.0722 |
| **Hypertension** | OR (95% CI): Ref. | 2.511 (1.151, 5.481) | 4.655 (2.533, 8.554) | 6.107 (1.737, 21.470) |
|  | P-value: - | 0.0208 | <0.0001 | 0.0048 |
|  | P Interaction: 0.9974 | 0.9974 | 0.9974 | 0.9974 |
| **Smoking** | OR (95% CI): Ref. | 2.904 (1.472, 5.731) | 3.612 (1.848, 7.060) | 8.734 (1.900, 40.150) |
|  | P-value: - | 0.0021 | 0.0002 | 0.0054 |
|  | P Interaction: 0.8847 | 0.8847 | 0.8847 | 0.8847 |

# Supplementary Table 3: Cancer Type Distribution in the Study Cohort

| ​Cancer Type | ​Number of Cases (n) | ​Percentage (%) |
| --- | --- | --- |
| Skin cancer (non-melanoma) | 676 | 22.8% |
| Breast cancer | 449 | 15.1% |
| Prostate cancer | 453 | 15.3% |
| Thyroid cancer | 60 | 2.0% |
| Melanoma | 176 | 5.9% |
| Bladder cancer | 74 | 2.5% |
| Cervical cancer | 193 | 6.5% |
| Colorectal cancer | 180 | 6.1% |
| Lung cancer | 70 | 2.4% |
| Uterine cancer | 116 | 3.9% |
| Ovarian cancer | 67 | 2.3% |
| Pancreatic cancer | 6 | 0.2% |
| Rectal cancer | 9 | 0.3% |
| Soft tissue sarcoma | 4 | 0.1% |
| Stomach cancer | 19 | 0.6% |
| Testicular cancer | 15 | 0.5% |
| Other tumors | 172 | 5.8% |
| Oral cancer | 15 | 0.5% |
| Nervous system tumors | 1 | 0.0% |
| Lymphoma | 59 | 2.0% |
| Kidney cancer | 56 | 1.9% |
| Leukemia | 32 | 1.1% |
| Liver cancer | 12 | 0.4% |
| Esophageal cancer | 14 | 0.5% |
| Gallbladder cancer | 1 | 0.0% |
| Laryngeal cancer | 10 | 0.3% |

# Supplementary Figure 1. Interaction effect forest plot of SII/GNRI subgroups across clinical variables.

#
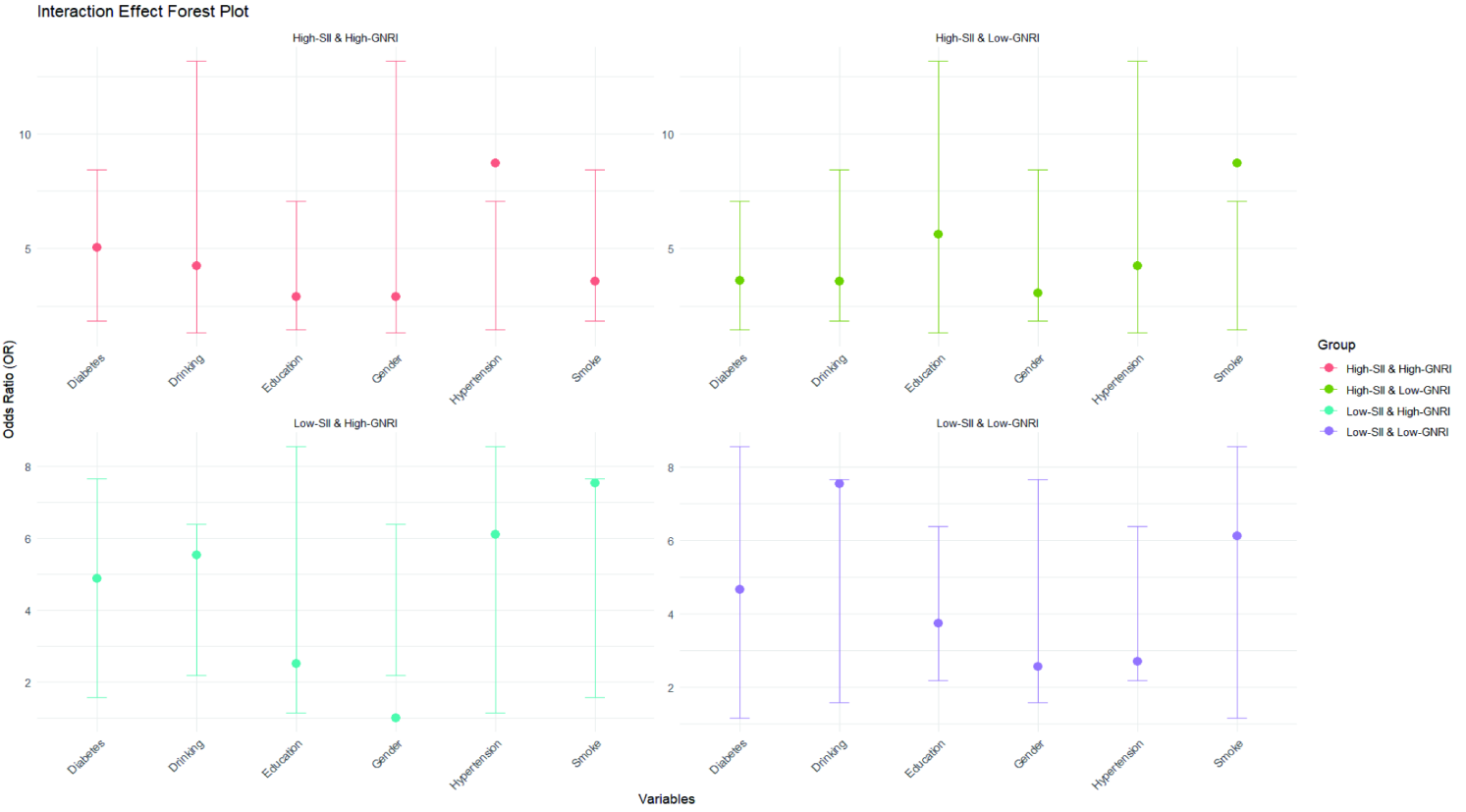


**Supplementary Figure 1 note**. This figure compares the odds ratios (ORs) of diabetes, alcohol consumption, education, gender, hypertension, and smoking across four SII/GNRI subgroups: High-SII/High-GNRI (red), High-SII/Low-GNRI (green), Low-SII/High-GNRI (cyan), and Low-SII/Low-GNRI (purple). The plot reveals significant interaction effects for gender (P = 0.048) and alcohol use (P = 0.072), where their impacts varied by SII/GNRI status. In contrast, diabetes, hypertension, and smoking showed consistent effects across all subgroups (P < 0.01) without significant interactions (P > 0.68). Education exhibited subgroup-specific effects but no interaction (P = 0.577). Error bars represent 95% confidence intervals, with OR values displayed on a logarithmic scale. The visualization highlights gender and alcohol as key modifiers of SII/GNRI effects, while other variables maintained independent associations.
